# Respiratory infections regulated blood cells IFN‐β‐PD‐L1 pathway in pediatric asthma

**DOI:** 10.1002/iid3.307

**Published:** 2020-05-12

**Authors:** Julia Kölle, Patricia Haag, Tytti Vuorinen, Kiefer Alexander, Manfred Rauh, Theodor Zimmermann, Nikolaos G. Papadopoulos, Susetta Finotto

**Affiliations:** ^1^ Department of Molecular Pneumology Friedrich‐Alexander‐Universität (FAU) Erlangen‐Nürnberg, Universitätsklinikum Erlangen Erlangen Germany; ^2^ Department of Virology University of Turku Turku Finland; ^3^ Department of Allergy and Pneumology, Children's Hospital Friedrich‐Alexander‐Universität (FAU) Erlangen‐Nürnberg, Universitätsklinikum Erlangen Erlangen Germany; ^4^ Allergy and Clinical Immunology Unit, 2nd Pediatric Clinic National and Kapodistrian University of Athens Athens Greece; ^5^ Division of Infection, Immunity & Respiratory Medicine University of Manchester Manchester UK

**Keywords:** human rhinovirus, IFNβ, PD‐L1, pediatric asthma

## Abstract

**Background:**

Respiratory infections, in general, and rhinovirus infection specifically are the main reason for asthma exacerbation in children and programmed cell death protein 1 ligand (PD‐L1) expression inhibits T cell responses.

**Objective:**

Could the interferon (IFN) type I expression in peripheral blood mononuclear cells (PBMCs) improve disease exacerbation in pediatric asthma?

**Results:**

Here we found increased level of *PD‐L1* messenger RNA (mRNA) in total blood cells isolated from preschool children with virus‐induced asthma, with lower percentage of forced expiratory volume in 1 second and with high serum levels of the C‐reactive‐protein.

**Conclusions and Clinical Relevance:**

These data indicate that, in the presence of infection in the airways of preschool children, worse asthma is associated with induced *PD‐L1* mRNA expression in blood cells. Further, type I IFN, IFN‐β, a cytokine that is involved in the clearance of infections, was found to be associated with a better lung function in asthmatic children. These data suggest that improving peripheral blood IFN type I expression in PBMCs in pediatric asthma could improve disease exacerbation due to suppressing PD‐L1 expression in blood cells.

## Graphical abstract

- Here we found increased level of programmed cell death protein 1 ligand (PD‐L1) messenger RNA (mRNA) in total blood cells isolated from preschool children with virus‐induced asthma, with lower percentage of forced expiratory volume in 1 second (FEV1%) and with high serum levels of the C‐reactive‐protein (CRP).
- Further, the activation of regulatory elements that induce interferon‐β (IFNβ), a cytokine that is involved in immunity of infections, was found to be associated with better lung function in asthmatic children.
- Finally, IFN‐β released by peripheral blood mononuclear cells (PBMCs) was found associated with an induced expression of PD‐L1 mRNA in control but no asthmatic children.

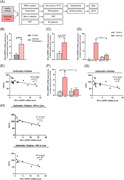

## 1 INTRODUCTION

The immune responses of the host to respiratory infections, in general, and to rhinovirus (RV) infection in particular, are associated with upregulation of type I interferon (IFN) pathways^1^, ^2^ in the airways and systemically in the blood cells.^3^ Deficient systemic IFN responses to respiratory infections have been observed in patients with noncontrolled asthma,^1^, ^2^, ^4^, ^5^ suggesting that type I IFN could be used to improve lung function in asthma. IFN response of the host can be suppressed by infectious agents by upregulation of programmed cell death protein 1 ligand (PD‐L1),^2^, ^6^ which then inhibit T cell proliferation via binding to programmed cell death protein 1 (PD1), considered as an immune checkpoint because it downregulates the immune responses.^7^

To analyze the influence of RV on IFN responses in asthma, we concentrated on the influence of human RV in the airways on IFN‐induced PD‐L1 in the peripheral blood cells of children with and without asthma.^8^, ^9^

## 2 METHODS

### 2.1 Human study PreDicta

In the European Study PreDicta (post‐infectious immune reprogramming and its association with persistence and chronicity of respiratory allergic diseases), we examined healthy and asthmatic preschool children at the age of 4 to 6 years in collaboration with the children hospital in Erlangen. The study in Erlangen was approved by the ethics committee of the Friedrich‐Alexander University Erlangen‐Nürnberg, Germany (Re‐No 4435) and it is registered in the German Clinical Trials Register (www.germanctr.de: DRKS00004914).

Two cohorts of preschool children (age 4‐6 years) with and without asthma were analyzed. The recruitment of the subjects, inclusion and exclusion criteria as well as the timescale for clinical visits and data collection were exactly described recently^1^, ^2^, ^5^, ^6^ along with the clinical aspects and characteristics and reported in other form in Tables 1 and 2.

**Table 1:** Demographic and clinical data of the healthy PreDicta cohort WP1‐UK‐ER analyzed at the baseline visit

Patient	Skin prick test^a^	Atopic dermatitis	Microbial swab result	FEV1% predicted^b^	PEF% predicted^b^	CRP, mg/L
208	n.d.	No	RV−	77	75	n.d.
211	n.d.	No	RV+	121	94	1.40
214	n.d.	No	RV+	110	94	0.29
215	n.d.	No	RV−	118	78	0.90
218	n.d.	No	RV+	111	92	0.68
219	n.d.	No	RV+	107	n.d.	0.48
220	negative	No	RV−	84	60	0.78
221	n.d.	No	RV+	n.d.	n.d.	0.26
222	n.d.	Yes	RV−	105	86	0.22
226	n.d.	No	RV+	109	93	1.25
227	n.d.	No	RV+	87	95	21.92
232	negative	No	RV+	100	70	0.76
233	n.d.	No	RV+	112	105	0.79
234	al	No	RV+	119	95	1.74
235	ca, f	No	RV+	113	75	n.d.
236	n.d.	No	RV−	111	101	0.11
237	negative	No	RV−	109	101	2.16
240	negative	No	RV+	92	74	0.64
241	negative	No	RV+	123	79	0.36
245	negative	No	RV−	121	106	0.51
246	negative	Yes	RV+	109	92	0.74
Average	Pos. = 9.5%	Yes = 9.5%	RV + = 66.7%	106.9 ± 2.9	87.6 ± 3.0	1.89 ± 1.12
	Neg. = 33.3%	No = 90.5%	RV− = 33.3%			

Abbreviations: CRP, C‐reactive‐protein; FEV1, forced expiratory volume in 1 s/forced vital capacity; PEF, peak expiratory flow; RV, rhinovirus.

^a^ al, *Alternaria* species; ca, cat; f, *Dermatophagoides* mix; n.d., not done.

^b^ Lung function results pre‐bronchodilation.

**Table 2:** Demographic and clinical data of the asthmatic PreDicta cohort WP1‐UK‐ER analyzed at the baseline visit

Patient	Asthma severity^a^	Phenotype^b^	Skin prick test^c^	Treatment	Atopic dermatitis	Microbial swab result	FEV1% predicted^d^	PEF% predicted^d^	CRP, mg/L
201	I	v	al, ca, g	Steroid	Yes	RV+	126	132	0.4
202	II	u	al, b, g	Steroid	Yes	RV+	111	–	/
203	II	u	ca	Steroid	No	RV−	95	80	1.22
204	II	a	al, am, ca, f, g	Steroid	Yes	RV−	128	127	0.31
205	I	u	ca	Steroid	No	RV−	102	86	2.13
206	I	u	al	Steroid	No	RV+	129	119	0.69
207	I	v	g	Steroid	Yes	RV−	143	117	0.13
209	II	v, a	g	Steroid	Yes	RV−	115	88	/
210	I	v	b, g	Nonsteroid	Yes	RV−	98	77	5.34
212	II	e, v	negative	Steroid	No	RV−	96	84	/
213	III	e	negative	Steroid	No	RV+	115	106	0.13
216	III	a, v	ca, f, g	Steroid	No	RV−	92	75	0.50
217	I	a, e, v	b, ca, f, g	Steroid	Yes	RV−	111	104	1.01
223	I	v	ca, f, g	Steroid	Yes	RV+	99	90	0.63
224	I	v	negative	Steroid	No	RV+	135	107	0.39
225	I	v	negative	Steroid	No	RV+	99	82	/
228	I	v	ca, f, g	Nonsteroid	No	RV−	88	65	0.37
229	I	v	al, b, ca, f, g	Nonsteroid	Yes	RV+	87	65	/
230	I	v	al, am, b, ca, f, g	Nonsteroid	Yes	RV+	101	86	0.69
231	I	v	b	Steroid	No	RV−	71	60	1.87
238	I	v	negative	Steroid	No	RV+	77	54	20.33
239	I	e	n.d.	Nonsteroid	No	RV+	98	92	0.55
242	II	a, e, v	al, b, ca, f, g	Steroid	No	RV+	81	99	0.64
243	II	v	negative	Steroid	No	RV+	69	53	2.90
Average	I = 62.5%	u = 16.7%	Pos. = 73.9%	Steroid = 79.2%	Yes = 41.7%	RV + = 54.2%	102.8 ± 4.0	89.0 ± 4.7	2.12 ± 1.05
	II = 29.2%	v = 70.8%	Neg. = 26.1%	Nonsteroid = 20.8%	No = 58.3%	RV− = 45.8%			
	III = 8.3%	a = 4.2%							
		e = 8.3%							

Abbreviations: CRP, C‐reactive‐protein; FEV1, forced expiratory volume in 1 s/forced vital capacity; PEF, peak expiratory flow; RV, rhinovirus.

^a^ I = Intermittent: FEV1 > 80%, MEF > 65%, symptom‐free interval >2 mo; II = mild persistent: FEV1 > 80%, MEF > 65%, symptom‐free interval <2 mo; III = moderate persistent: FEV1 < 80%, MEF < 65%, symptoms several days a week; IV = severe persistent: FEV1 < 60%, symptoms during the day and night.

^b^ v, virus‐induced; a, allergen‐induced; e, exercise‐induced; u, unresolved.

^c^ al, *Alternaria* species; am, ambrosia; b, birch; ca, cat; f, *Dermatophagoides* mix; g, grass pollen mix; n.d., not done.

^d^ Lung function results pre‐bronchodilation.

For gene expression analysis, we isolated messenger RNA (mRNA) from total blood cells of the children as previously described and performed quantitative real‐time polymerase chain reaction (PCR) as described below.^1^ The levels of C‐reactive‐protein (CRP) in the serum samples of the children were measured by turbidimetry on a Roche Integra 800 Analyzer (CRPL2 reagent, limit of detection 1.0 mg/L, interday CV 1.4% [8.1 mg/L]; Roche Diagnostics, Basel, Switzerland). The detection of RV in nasopharyngeal swab obtained from the children was performed at the Department of Virology, University of Turku (Finland). The description of this procedure is already published in detail elsewhere.^1^

### 2.2 FEV1 and PEF

The percentage of forced expiratory volume in 1 second (FEV1), forced vital capacity (FVC), and peak expiratory flow (PEF) were measured at baseline visit (B0) by using spirometry. After a period of normal breathing, the participant should inhale maximal, directly followed by maximal and fast exhalation. The volume exhaled in 1 second is FEV1. The total exhaled volume is FVC. The ratio FEV1/FVC is stated as FEV1%. The PEF is defined as the largest expiratory flow, which is achieved with a maximum forced effort after maximum inspiration.

### 2.3 Human RNA isolation from Tempus Tubes and quantitative real‐time polymerase chain reaction

At baseline visit, whole blood was collected in Tempus® Blood RNA Tubes (Life Technologies™, GmbH, Darmstadt, Germany) and RNA was extracted with the MagMax for Stabilized Blood Tubes RNA Isolation Kit. For reverse transcription of RNA (1 µg), we used the first strand complementary DNA (cDNA) synthesis kit for RT‐PCR (MBI Fermentas, St. Leon‐Rot, Germany). The resulting template cDNA was then amplified by quantitative real‐time PCR (qPCR) using SoFast EvaGreen Supermix (Bio‐Rad Laboratories, München, Germany). The qPCR itself was performed in a CFX96 Touch Real‐Time PCR Detection System (Bio‐Rad Laboratories) with a cycle of 2 minutes at 98°C, 50 cycles of 5 seconds at 95°C, 10 seconds at 60°C, followed by 5 seconds at 65°C and 5 seconds at 95°C. The primer sequences used for the real‐time PCR are listed in Table S1. The mRNA of the genes of interest was normalized using the housekeeping gene hypoxanthine guanine phosphoribosyl transferase (*HPRT*).

### 2.4 Isolation of peripheral blood mononuclear cells, in vitro cell culture, and analysis of the cell supernatants

At the time of recruitment (baseline visit), peripheral blood mononuclear cells (PBMCs) were isolated from heparinized blood with Ficoll using density centrifugation. After isolation, PBMC numbers were adjusted to a concentration of 10^6^ viable cells/mL in complete culture medium. For cell culture, Roswell Park Memorial Institute 1640 medium supplemented with 25 mmol/L HEPES (Gibco, Invitrogen, Darmstadt, Germany) was used. Furthermore, 100 IU/mL penicillin, 100 µg/mL streptomycin, 50 µmol/L β‐mercaptoethanol, 1% l‐glutamine (200 mmol/L), 1% MEM Vitamin, 1% nonessential amino acids, 1% sodium pyruvate, and 10% fetal bovine serum were added (complete culture medium); these reagents were purchased from Sigma‐Aldrich (Steinheim, Germany). The PBMCs were cultured in complete culture medium for 24 hours at 37°C and 5% CO_2_, whereby parts of them were challenged in vitro with 10 µg/mL PHA (Sigma‐Aldrich) or with RV (RV1b). The growth of RV1b and the description of the RV1b infection itself have been published previously in detail elsewhere.^1^

Human IFNβ and interleukin 10 (IL‐10) was detected in the cell‐culture supernatants by using IFNβ ELISA kit from PeproTech (Hamburg, Germany) and IL‐10 OptEIA™ sandwich ELISA kit from BD Bioscience (Heidelberg, Germany), respectively, according to the manufacturer's protocol.

### 2.5 Statistical analysis

Statistical analysis was performed using Prism (version 7) for Windows (GraphPad, La Jolla, CA). Differences were evaluated for significance by using the two‐tailed Student *t* test or ordinary one‐way analysis of variance to generate *P*‐value data (*P ≤ .05, **P ≤ .01, ***P ≤ .001, ****P ≤ .0001) for all data. Unless otherwise indicated, data are presented as mean ± SEM.

## 3 RESULTS

### 3.1 PD‐L1 is induced in blood cells of preschool asthmatic children with a virus‐induced asthma phenotype and associated with the presence of rhinovirus in their airways

**Figure 1:**
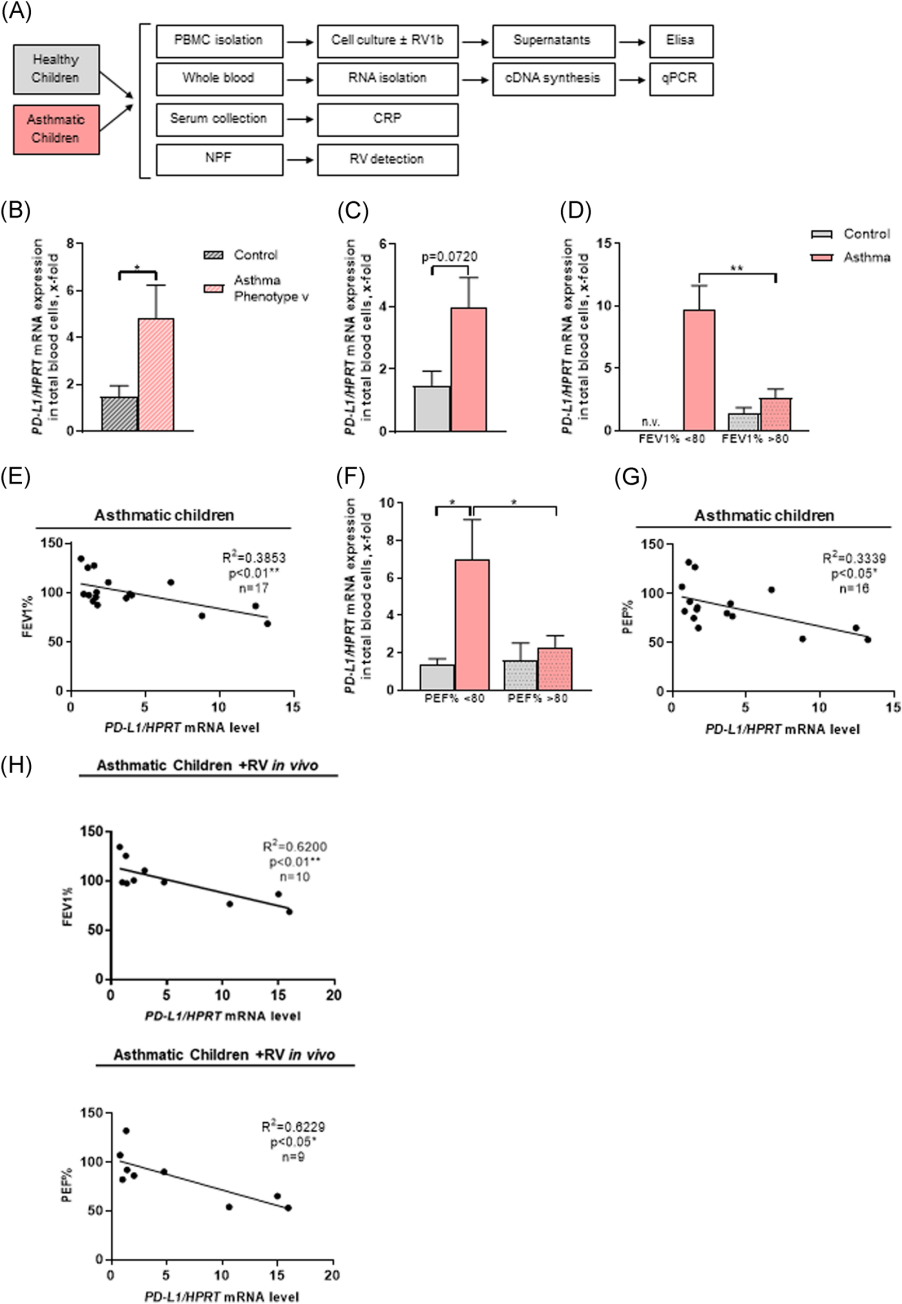
Regulation of programmed cell death protein 1 ligand (*PD‐L1*) messenger RNA (mRNA) level in blood cells of preschool children. A, Experimental design of the blood and nasopharyngeal fluid analysis of the healthy (n = 21) and asthmatic (n = 24) preschool children of the PreDicta cohort in Erlangen. B, *PD‐L1/*hypoxanthine guanine phosphoribosyl transferase (*HPRT*) mRNA expression in total blood cells of healthy and asthmatic children with a virus‐induced (v) asthma phenotype at the baseline visit (n = 10/11). C, *PD‐L1/HPRT* mRNA expression in total blood cells of healthy and asthmatic children (n = 10/17). D, *PD‐L1/HPRT* mRNA expression in total blood cells of healthy and asthmatic children subdivided according to their forced expiratory volume in 1 second percentage (FEV1%) at the baseline visit (n = 0/2/9/15). E, Correlation of the *PD‐L1/HPRT* mRNA level in total blood cells of asthmatic children with the FEV1% at the baseline visit. F, *PD‐L1/HPRT* mRNA expression in total blood cells of healthy and asthmatic children subdivided according to their peak expiratory flow percentage (PEF%) at the baseline visit (n = 5/6/5/10). G, Correlation of the *PD‐L1/HPRT* mRNA level in total blood cells of asthmatic children with the PEF% at the baseline visit. H, Correlation of the *PD‐L1/HPRT* mRNA level in total blood cells of asthmatic children with rhinovirus (RV) in their airways with the FEV1% (top) and PEF% (bottom) at the baseline visit. Data are presented as means ± SEM. Two‐tailed Student *t* test (b and c) or ordinary one‐way analysis of variance (ANOVA; d and f) was used to calculate statistical significance. *P ≤ .05; **P ≤ .01, ***P ≤ .001, ****P ≤ .0001

We recently described that acute in vitro infection of PBMCs from preschool children with and without asthma with RV, a single‐stranded RNA picornavirus, is associated with the upregulation of IFN‐regulated genes like STAT1, STAT2, and IFN regulatory factor 1.^1^, ^2^ Moreover, paradoxically, IFNγ upregulates also PD‐L1, a factor involved in silencing/exhausting of activated T cells by ligating PD1 on the surface of T cells.^10^ Consistently, we found that acute RV infection ex vivo induced PD‐L1 and CTLA4 in the PBMCs of asthmatic children.^6^ We thus wanted to follow up these in vitro observations in the two cohorts of our study and analyzed 21 control children and 24 children with asthma (Figure 1A). The clinical data of these cohorts of children were recently reported^2^, ^5^, ^6^ and are summarized in Tables 1 and 2. By looking at the PD‐L1 mRNA expression in blood, we found that *PD‐L1* mRNA expression was induced in children with a virus‐induced asthma phenotype (in accordance to PRACTALL guidelines 2008^11^) compared to healthy control children (Figure 1B). Children with this asthma phenotype shows symptom‐free periods, whereas the most common precipitating factor are colds by respiratory viruses, like human RV.^11^

Furthermore, by trend, we observed an induction of PD‐L1 mRNA in the blood cells of asthmatic children as compared to control children (Figure 1C). We next analyzed PD‐L1 expression after allergen and RV challenge. Considering the presence of RV (+RV) in the airways, we found that, by trend, asthmatic children with RV in the airways, have an increased PD‐L1 mRNA expression in total blood cells (Figure S1a). This is also associated with increased expression of the low‐density lipoprotein receptor (Figure S1b), which is one of the main receptors used by the viruses, especially for RV1b, to entering the cells.

### 3.2 PD‐L1 is upregulated in blood cells of asthmatic children with increased bronchoconstriction

We then asked if the lung function, especially the FEV1% as well as the PEF% (predicted), of the cohorts would correlate with increased PD‐L1 expression in blood. The FEV1/FVC ratio (FEV1%) is a calculated ratio used in the diagnosis of obstructive and restrictive lung disease. It represents the proportion of a person's vital capacity that they are able to expire in the first second of forced expiration (FEV1) to the full, FVC. The result of this ratio is expressed as FEV1%.^12^ Lower values of FEV1% represent airway obstruction. In our cohort of children with asthma, but not in control children, we found a PD‐L1 induction in children with higher bronchoconstriction (Figure 1D) and an inverse correlation between PD‐L1 and FEV1% (Figure 1E), indicating that worse asthma is associated with induction of PD‐L1 mRNA in blood cells of children with asthma. We then further investigated the role of another lung function parameter, the PEF% value (Figure 1F,G). The PEF% is defined as the largest expiratory flow, which is achieved with a maximum forced effort after maximum inspiration and is used as a control parameter during asthma therapy. Similar to the FEV1%, we found a significant PD‐L1 induction in children with worse asthma (Figure 1F) as well as an inverse correlation between PD‐L1 expression and the PEF% (Figure 1G). We also found that increased PD‐L1 mRNA expression correlated with reduced FEV1% and PEF% (Figure 1H), indicating that asthmatic preschool children with RV colonization in the airways have worse respiratory function associated with PD‐L1 induction in their PBMCs. By contrast, healthy control children with and without RV in the airways as well as in asthmatic children without RV colonization in the airways no correlation between FEV1% or PEF% and PD‐L1 was observed (Figure S1c,d).

### 3.3 Interferon‐β correlated with better lung function in asthmatic children

**Figure 2:**
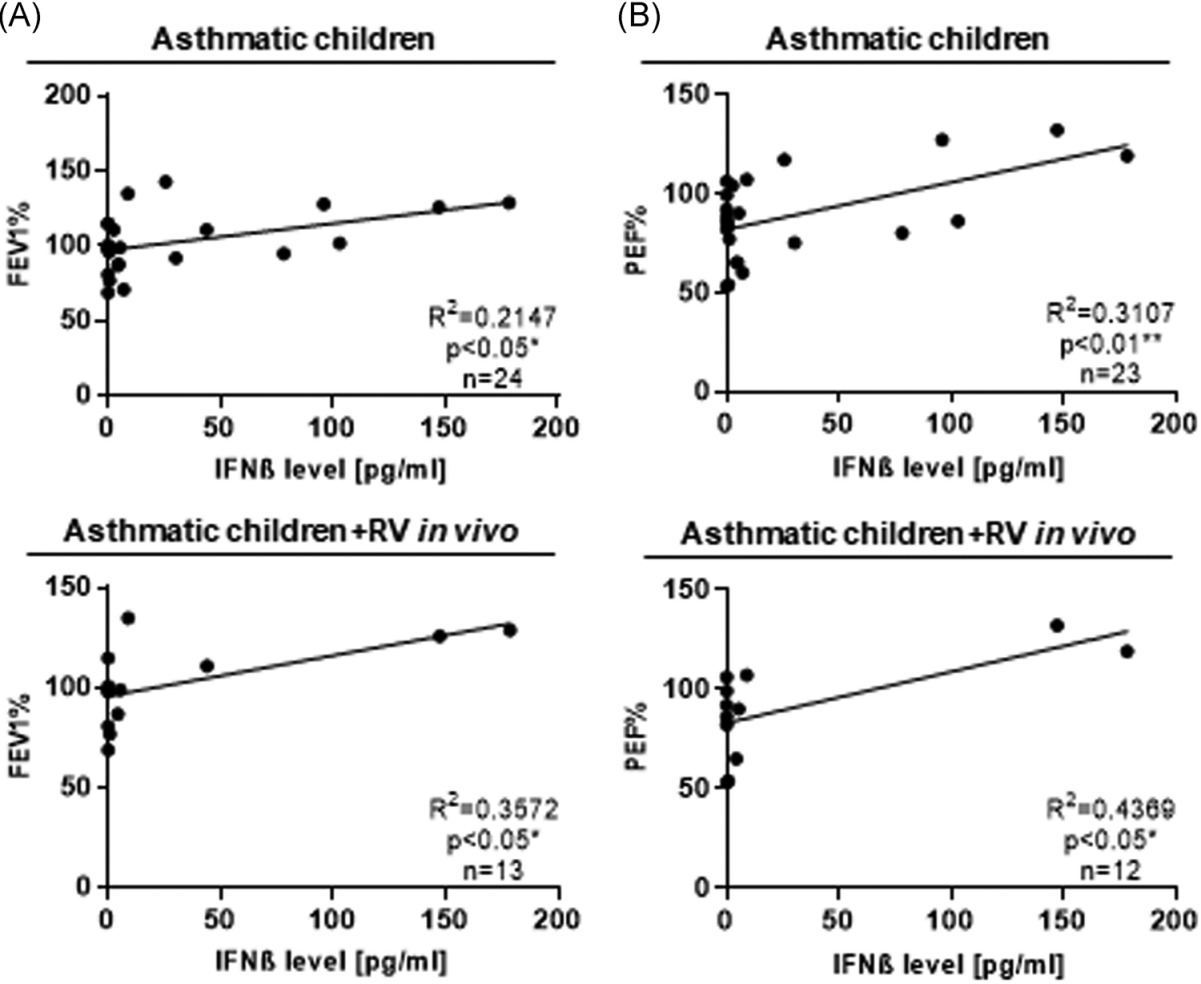
Interferon‐β (IFNβ) correlated with better lung function in asthmatic children. A,B, Correlation of the IFNβ level, measured in the supernatants of the untreated peripheral blood mononuclear cell (PBMC) culture and the respective FEV1% (i) and PEF% (j) of asthmatic children with and without RV in their airways. *P ≤ .05; **P ≤ .01, ***P ≤ .001, ****P ≤ .0001. FEV1, forced expiratory volume in 1 second; PEF, peak expiratory flow; RV, rhinovirus

We next reasoned that in the case of asthma induced by infections, especially RV infections, IFN‐type I and specifically IFNβ might be of importance.^13^ Thus, we next analyzed the IFNβ level in cell culture supernatants of untreated PBMCs from healthy and asthmatic children with and without RV in the airways (Figure 2A) as well as after a restimulation with RV1b in vitro (Figure S2a,b) and correlated them with their FEV1% and PEF% (Figure 2A,B; Figure S2c‐e and S3). Here we found that, only asthmatic children and especially asthmatic children with RV in their upper airways show a direct correlation between the IFNβ level and the FEV1% and PEF%, respectively, indicating that a subpopulation of children could respond to RV infection with IFNβ production.

### 3.4 PD‐L1 levels correlated with IFNβ production in healthy but not in asthmatic children

**Figure 3:**
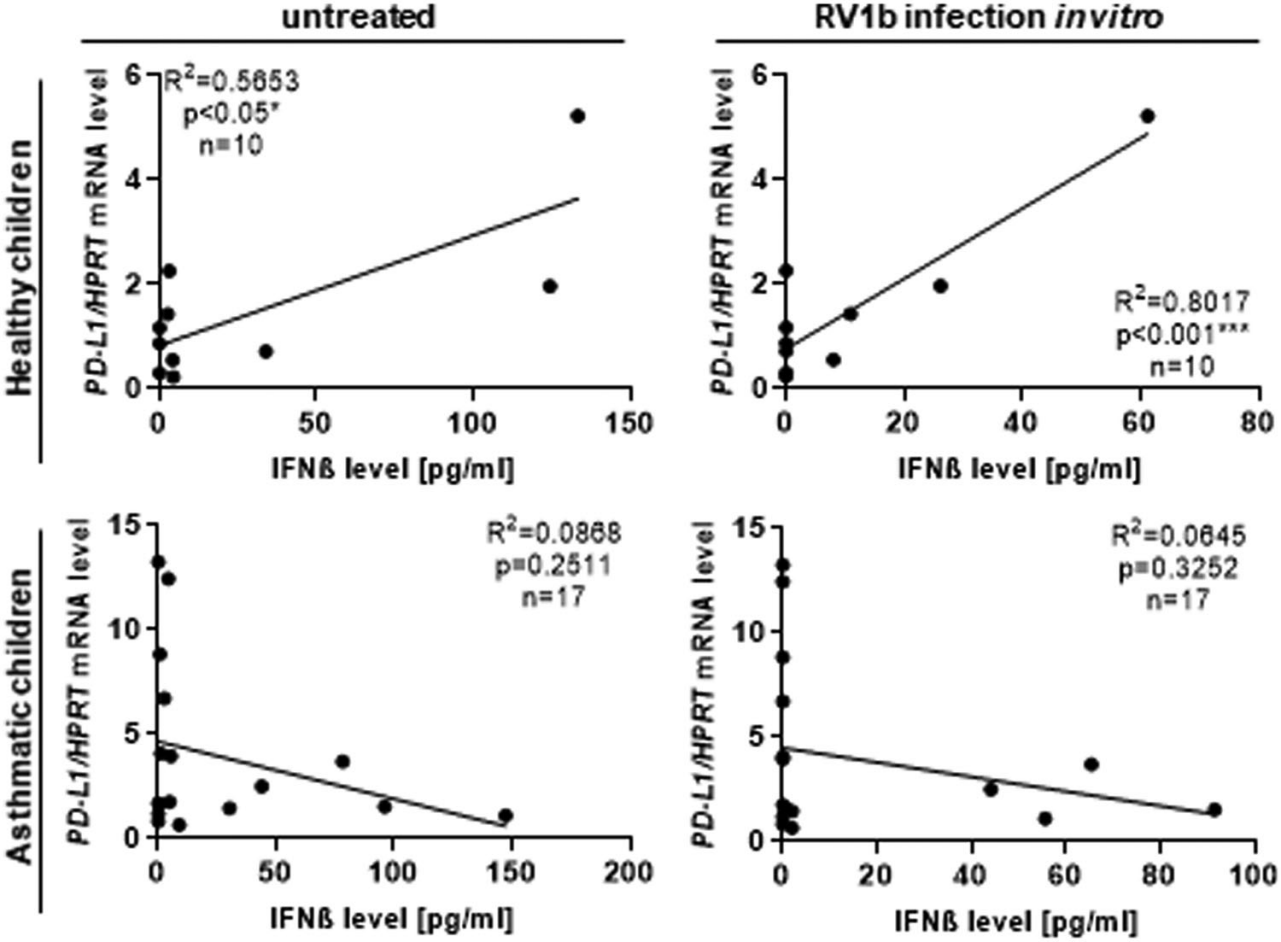
IFNβ correlated with *PD‐L1* mRNA level in control children but not in asthmatic children. Correlation of the *PD‐L1/HPRT* mRNA level in total blood cells with the IFNβ level, measured in the supernatants of the respective untreated and with RV1b restimulated PBMC culture, of healthy and asthmatic children. *P ≤ .05, **P ≤ .01, ***P ≤ .001, ****P ≤ .0001. IFNβ, interferon‐β; mRNA, messenger RNA; PBMC, peripheral blood mononuclear cell; PD‐L1, programmed cell death protein 1 ligand

Since it is known that IFN induces PD‐L1,^14^ we correlated the IFNβ expression in the supernatants of untreated and with RV1b restimulated PBMCs and the PD‐L1 expression in total blood cells and found a direct correlation in control children, but not in asthmatic children (Figure 3 and Figure S4a,b). These data indicate that IFNβ is associated with PD‐L1 in control children and that asthmatic children have a disturbed IFNβ‐mediated PD‐L1 induction.

### 3.5 PD‐L1 is upregulated in blood cells of asthmatic children with high C‐reactive protein serum levels and correlated with RV in the airways

**Figure 4:**
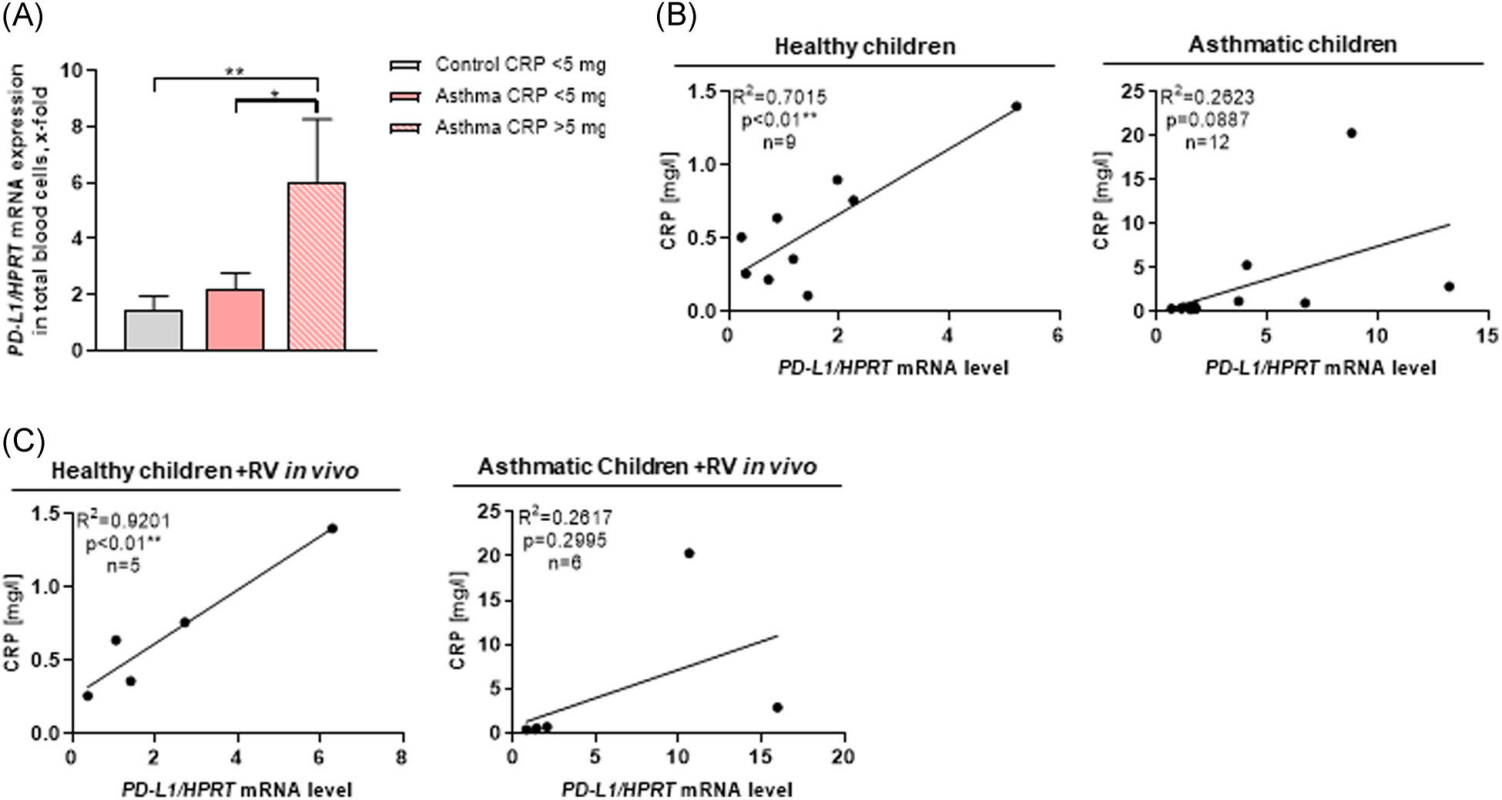
PD‐L1 is upregulated in blood cells of asthmatic children with high C‐reactive protein (CRP) serum levels and correlated with RV in the airways. A, *PD‐L1/HPRT* mRNA expression in total blood cells of healthy and asthmatic children subdivided according to their CRP serum level (n = 9/10/2). B,C, Correlation of the *PD‐L1/HPRT* mRNA level in total blood cells with the CRP serum level subdivided in healthy and asthmatic children with and without RV in their airways. Data are presented as means ± SEM. Ordinary one‐way ANOVA was used to calculate statistical significance. *P ≤ .05, **P ≤ .01, ***P ≤ .001, ****P ≤ .0001. ANOVA, analysis of variance; *HPRT*, hypoxanthine guanine phosphoribosyl transferase; mRNA, messenger RNA; PD‐L1, programmed cell death protein 1 ligand; RV, rhinovirus

We next reasoned that not only RV but also other infection or inflammatory agents could cause PD‐L1 induction in asthmatic children. We thus next looked at the CRP level in serum of our cohorts of children. CRP binds to the phosphocholine expressed on the surface of dead or dying cells and some bacteria and leading to the activation of the complement system and promotion of phagocytosis by macrophages.^15^ Higher levels are found in inflammation, viral infections (10‐40 mg/L), active bacterial infection (40‐200 mg/L), severe bacterial infections, and burns (>200 mg/L).^16^ We considered high CRP levels as an indicator of ongoing infection and inflammation and found that children with asthma and a CRP value over 5 mg/L had a significantly higher PD‐L1 mRNA expression in total blood cells as compared to the control children (Figure 4A). Moreover, in both healthy and asthmatic children, CRP was found to be associated with high PD‐L1 levels in the serum (Figure 4B and Figure S4c). Finally, in the presence of RV in the airways, CRP correlated with PD‐L1 expression in healthy children (Figure 4C). Taken together, these data suggest the presence of induced PD‐L1^+^ cells in the blood of asthmatics with worse asthma and ongoing inflammation and infection.

## 4 DISCUSSION

Here we found increased PD‐L1 mRNA levels in total blood cells isolated from preschool asthmatic children with a virus‐induced asthma phenotype, lower FEV1% and with high CRP serum levels, indicating that worse asthma, in the presence of infections in the airways, is associated with induced *PD‐L1* mRNA expression. IFNβ, released by PBMCs in preschool children with HRV infected airways was found to correlate with improved lung function, both in control and asthmatic children. However, although in control children IFNβ directly correlated with *PD‐L1* mRNA expression, in asthmatic children this correlation was lost in peripheral blood.

PD‐L1 has been associated with hepatitis B infections.^17^ In this case, the use of anti‐PD‐L1 inhibitors was suggested to improve natural killer T cell function resulting in inhibition of virus replication. This mechanism seems to be similar to a described mechanism in lung cancer where anti‐PD‐L1 antibody treatment results in ameliorated antitumour immune response.^14^ Here we found that PD‐L1 mRNA was induced in association with higher levels of the infection marker CRP in the periphery but not with RV in the airways. In addition, PD‐L1 mRNA did not directly correlated with IFN‐β release in the peripheral blood of asthmatic children, indicating a possible therapeutical IFN‐mediated therapy for these asthmatic children. Further, we recently reported that these asthmatic children have prevalent Gram‐negative colonization in the airways which are associated with induction of IFN‐β release in the airways in their nasal pharyngeal fluid.^5^ Thus it is possible that the direct correlation found between CRP and PD‐L1 relate to the presence of Gram‐negative bacteria in the airways of these children.

Taken together, these data reveal that the host respond to infection with release of IFNβ in blood cells. The infectious agent then redirects this response by upregulating PD‐L1, which inhibits the immune system. In asthma, there seems to be a therapeutical possibility to use IFN type 1 to improve lung function without inducing PD‐L1, thus keeping activated anti‐infection immune responses.

## CONFLICT OF INTERESTS

The authors declare that there are no conflict of interests.

## AUTHOR CONTRIBUTIONS

JK is the major investigator of this study and PH contributed to the children analysis. SF contributed to the design of this study, supervised this work, and wrote the manuscript. TV did the respiratory virus analysis in the nasal pharyngeal fluid of the children analyzed in this study. AK and TZ are the pediatricians that saw most of the children in Predicta WP1‐UKER and made the medical diagnosis. MR did the CRP analysis. NP designed the WP1 project Predicta and was the coordinator of Predicta.

## Supporting information

Supporting information

Supporting information

Supporting information

Supporting information

Supporting information

Supporting information

## Data Availability

The data that support the findings of this study are available on request from the corresponding author. The data are not publicly available due to privacy or ethical restrictions.
