# Supplementary figures and images for: Respiratory infections regulated blood cells IFN‐β‐PD‐L1 pathway in pediatric asthma
Source: Immun Inflamm Dis. 2020 May 12;8(3):310–9. doi: 10.1002/iid3.307 (PMC7416032; doi:10.1002/iid3.307)

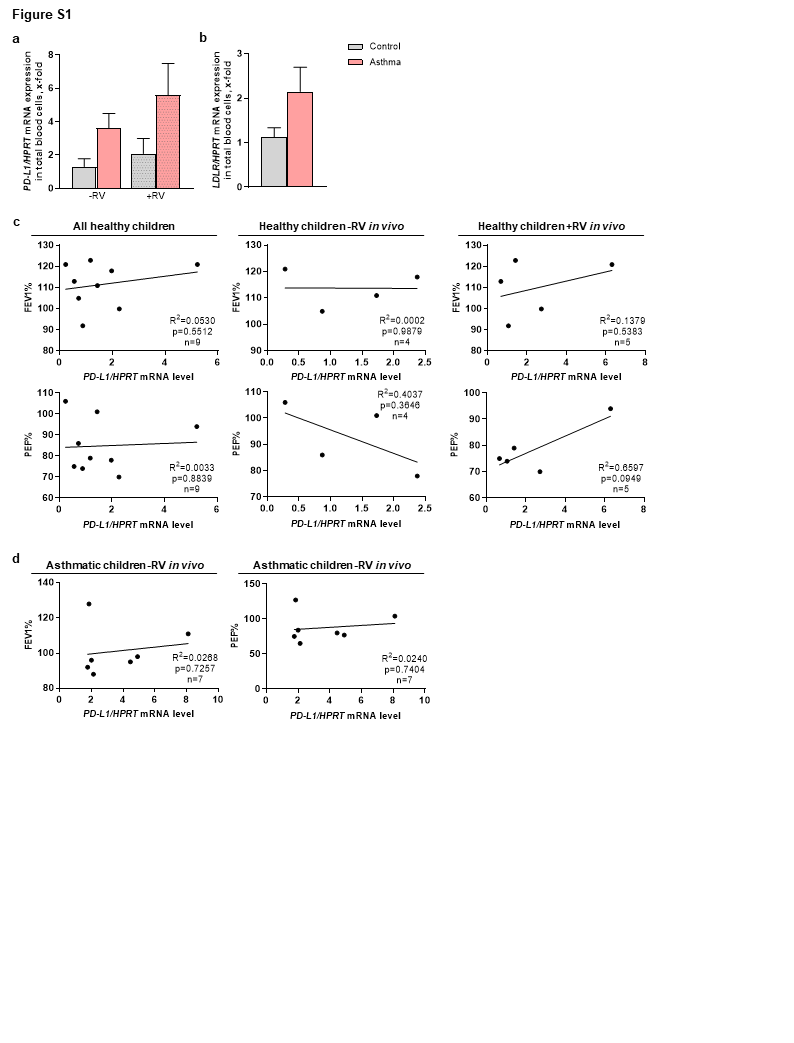

Supplement: Supplementary file 1 — Supporting information [file IID3-8-310-s001.TIF]

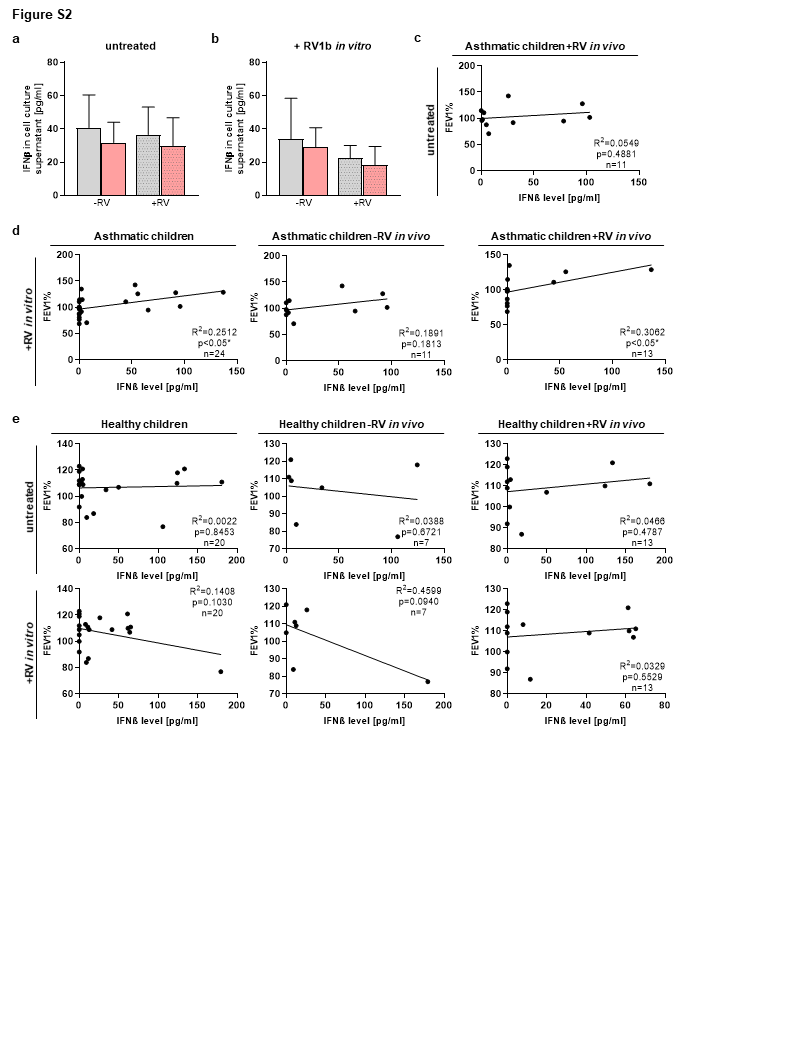

Supplement: Supplementary file 2 — Supporting information [file IID3-8-310-s002.TIF]

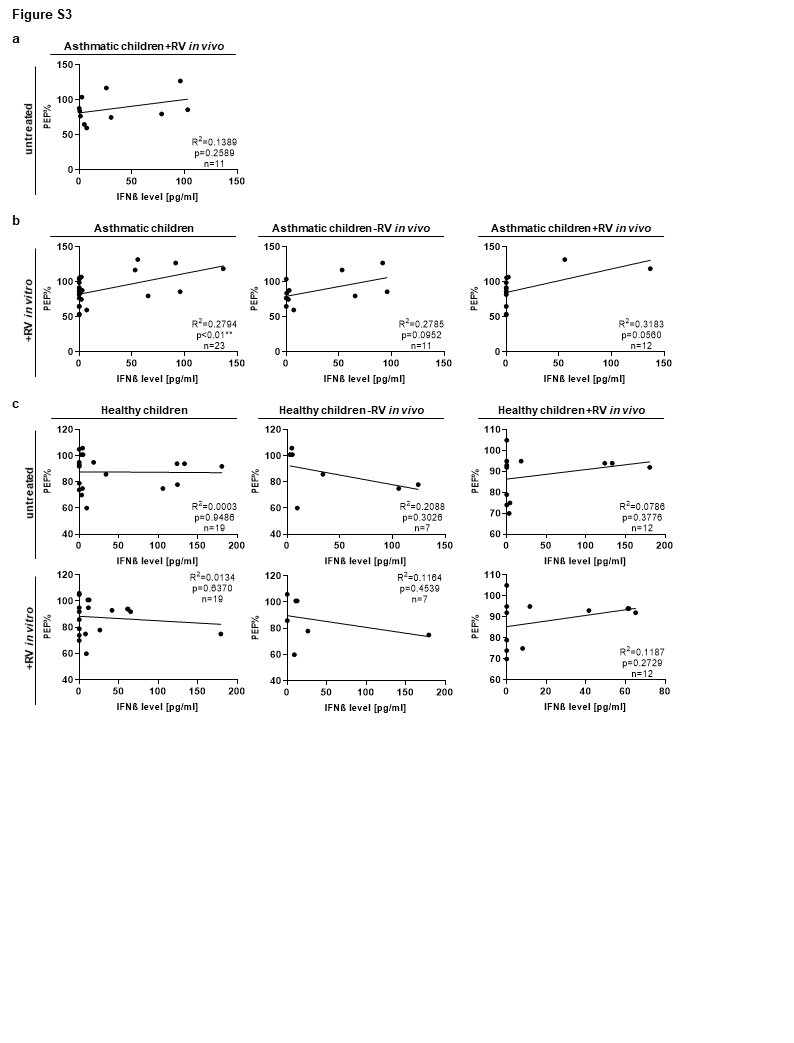

Supplement: Supplementary file 3 — Supporting information [file IID3-8-310-s003.TIF]

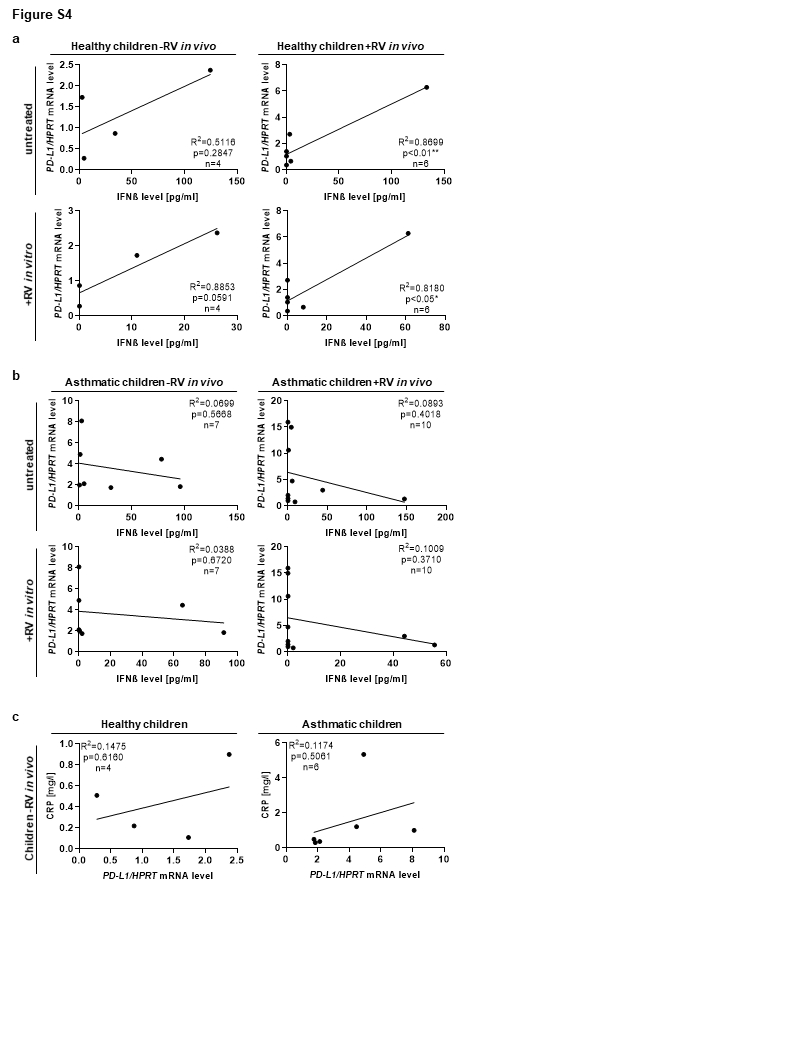

Supplement: Supplementary file 4 — Supporting information [file IID3-8-310-s004.TIF]

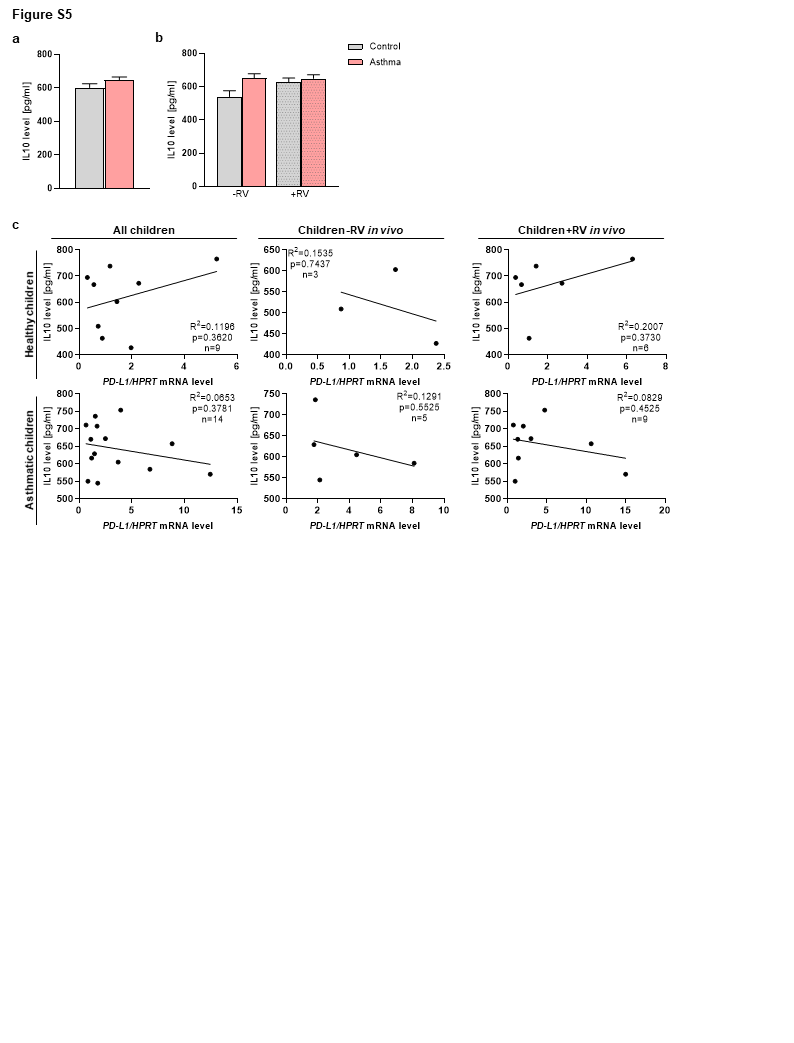

Supplement: Supplementary file 5 — Supporting information [file IID3-8-310-s005.TIF]
