# Supplementary material for: Respiratory infections regulated blood cells IFN‐β‐PD‐L1 pathway in pediatric asthma
Source: Immun Inflamm Dis. 2020 May 12;8(3):310–9. doi: 10.1002/iid3.307 (PMC7416032; doi:10.1002/iid3.307)
Supplement: Supplementary file 6 — Supporting information [file IID3-8-310-s006.doc]

**Respiratory infections regulated blood cells IFN-β-PD-L1 pathway in pediatric asthma**

Julia Kölle^1^, Patricia Haag^1^, Tytti Vuorinen^2^, Kiefer Alexander^3^, Manfred Rauh^3^,Theodor Zimmermann^3^, Nikolaos G. Papadopoulos^4,5^ and Susetta Finotto^1*^

^1^Department of Molecular Pneumology, Friedrich-Alexander-Universität (FAU) Erlangen-Nürnberg, Universitätsklinikum Erlangen, 91052 Erlangen, Germany

^2^Department of Virology, University of Turku, Kiinamyllynkatu 13, 20520 Turku, Finland

^3^Children’s Hospital, Department of Allergy and Pneumology, Friedrich-Alexander-Universität (FAU) Erlangen-Nürnberg, Universitätsklinikum Erlangen, 91054 Erlangen, Germany

^4^Allergy and Clinical Immunology Unit, 2nd Pediatric Clinic, National and Kapodistrian University of Athens, 11527 Athens, Greece

^5^Division of Infection, Immunity & Respiratory Medicine, University of Manchester, M13 9PL Manchester, UK

*** Correspondence:**Prof. Dr. Dr. Susetta Finotto

Universitätsklinikum Erlangen

Abt. Molekulare Pneumologie

Hartmannstraße 14

91052 Erlangen

Phone: +49-9131-85-42454

Mail: susetta.finotto@uk-erlangen.de

http://www.molekulare-pneumologie.uk-erlangen.de

Keywords: Human Rhinovirus, PD-L1, IFNβ, pediatric asthma.

**SUPPLEMENTARY FIGURE LEGENDS**

**Supplementary Figure 1. Regulation of PD-L1 mRNA level in the blood cells of preschool children. (a)** *PD-L1/HPRT* mRNA expression in total blood cells of healthy and asthmatic children with and without rhinovirus (RV) infection in their airways (n=4/7/6/10). **(b)** *LDLR/HPRT* mRNA expression in total blood cells of healthy and asthmatic children at the baseline visit (n=10/15). **(c, d)** Correlation of the PD-L1/HPRT mRNA level in total blood cells with the FEV1% and the PEF% at the baseline visit of healthy **(c)** and asthmatic **(d)** children with and without Rhinovirus in the airways. Data are presented as means ± SEMs. Ordinary one-way ANOVA (a) or two-tailed student *t* test (b) was used to calculate statistical significance. * p ≤ 0.05; ** p ≤ 0.01, *** p ≤ 0.001, **** p≤ 0.0001.

**Supplementary Figure 2. Regulation of the IFNβ level in the blood cells of preschool children. (a, b)** IFNβ level in the cell culture supernatant of untreated **(a)** and with RV1b re-stimulated **(b)** PBMC cell culture in healthy and asthmatic children with and without Rhinovirus in the airways (n=7/11/14/13). **(c-e)** Correlation of the IFNβ level, measured in the supernatants of the respective untreated and with Rhinovirus 1b re-stimulated PBMC culture, and the FEV1% of healthy and asthmatic children with and without Rhinovirus in the airways. Data are presented as means ± SEMs. Ordinary one-way ANOVA was used to calculate statistical significance. * p ≤ 0.05; ** p ≤ 0.01, *** p ≤ 0.001, **** p≤ 0.0001.

**Supplementary Figure 3. Correlation between the IFNβ level in the blood cells of preschool children and lung function. (a-c)** Correlation of the IFNβ level, measured in the supernatants of the respective untreated and with Rhinovirus 1b re-stimulated PBMC culture, and the PEF% of healthy and asthmatic children with and without Rhinovirus in the airways. * p ≤ 0.05; ** p ≤ 0.01, *** p ≤ 0.001, **** p≤ 0.0001.

**Supplementary Figure 4. Correlation between PD-L1 mRNA level with the IFNβ cell culture supernatant and the CRP serum level in healthy and asthmatic children. (a, b)** Correlation of the PD-L1/HPRT mRNA level in total blood cells with the IFNβ level, measured in the supernatants of the respective untreated and with Rhinovirus 1b re-stimulated PBMC culture, of healthy and asthmatic children with and without Rhinovirus in the airways. **(c)** Correlation of the PD-L1/HPRT mRNA level in total blood cells with the CRP serum level subdivided in healthy and asthmatic children without Rhinovirus in their airways. * p ≤ 0.05; ** p ≤ 0.01, *** p ≤ 0.001, **** p≤ 0.0001.

**Supplementary Figure 5. Regulation of the IL-10 level in the blood cells of preschool children. (a, b)** IL-10 level in the cell culture supernatant of PHA treated PBMC of healthy and asthmatic children with and without Rhinovirus in the airways (n=18/20 (a), n=5/8/13/12 (b)). **(c)** Correlation of the *PD-L1/HPRT* mRNA level in total blood cells of healthy and asthmatic children with and without Rhinovirus in the airways with the IL-10 level, measured in the supernatants of the respective PHA treated PBMC culture. Data are presented as means ± SEMs. Two-tailed student *t* test (a) or ordinary one-way ANOVA (b) was used to calculate statistical significance. * p ≤ 0.05; ** p ≤ 0.01, *** p ≤ 0.001, **** p≤ 0.0001.

**Table S1:** **Sequences of human and murine primers for qPCR used in this study.**

| **Target** | **Sequence forward primer** | **Sequence reverse primer** |
| --- | --- | --- |
| *hHPRT* | 5´-TGA CAC TGG CAA AAC AAT GCA-3´ | 5´-GGT CCT TTT CAC CAG CAA GCT-3´ |
| *hLDLR* | 5´-CCG TAA GGA CAC AGC ACA CA-3´ | 5´- CCC AGA GCT TGG TGA GAC ATT-3´ |
| *hPDL1* | 5´-AGCAAAGTGATACACATTTTGGAG-3´ | 5´-CCCCGATGAACCCCTAAACC-3´ |
